# A dataset on body composition, strength and performance in older adults

**DOI:** 10.1016/j.dib.2019.105103

**Published:** 2020-01-03

**Authors:** Arindam RoyChoudhury, Chenghuiyun Xu

**Affiliations:** Division of Biostatistics and Epidemiology, Department of Healthcare Policy and Research, Weill Cornell Medicine, Cornell University

**Keywords:** Frailty, Sarcopenia, Body composition, Physical activity, Grip strength

## Abstract

This article presents a dataset of body composition, strength and performance measurements in older adults; the data were collected as part of Rancho Bernardo Study (RBS), a longitudinal observational cohort study. All community dwelling adults in Rancho Bernardo, California between 1972 and 1974 were eligible for participation in the study. A subset of the participants returned every four years for subsequent visits. The dataset in this publication consists of some of the measures taken in Visits 7–10, for 1466 subjects who had at least one of these measures taken. We analysed the data with a feed-forward loop model fitted by structural equation modelling. The data can be valuable for modelling and extracting further information on how body composition, strength and performance affect each other over a long period of time. The data are analysed and interpreted in the research article RoyChoudhury et al., 2019.

**Table:** Specifications Table

Subject	Geriatrics and Gerontology
Specific subject area	Mechanism of development of frailty
Type of data	Table
How data were acquired	Body composition measures were measured using fan-beamed dual energy x-ray absorptiometry (DXA) using the model Hologic 2000 (Hologic, Inc., Bedford, MA, USA). Grip strength was measured using a handheld dynamometer (Sammons Preston Rolyan, Bolingbrook, IL, USA); for each subject, the grip strength of each hand was measured twice, and the maximum value from each hand was recorded. In this dataset, we have only included the average of the maximum grip strengths of the two hands. For the timing in Timed “Up and Go” test (TUG), the subjects were asked to rise from a chair, walk 3 m, and return to a sitting position. This was performed twice by each subject, and recorded to the tenth of a second. In this dataset, we have only included the average of the two TUG measures for each subject.
Data format	Raw Analysed Filtered
Parameters for data collection	All community dwelling adults in living in a California community (Rancho Bernardo) between 1972 and 1974 were eligible for enrolment [1]. A subset of the subjects returned every four years for follow up visits [2]. This dataset contains of some of the measurements from Visits 7–10.
Description of data collection	All community dwelling adults from Rancho Bernardo, California were invited to enrol. At the first visit, demographic information, physical characteristics, personal and family medical and health history were collected. A subset of the enrolees continued to return for follow up visits every four year.
Data source location	Institution: University of California, San Diego City/Town/Region: Rancho Bernardo Country: USA
Data accessibility	Tables are available with this article. The dataset is available through the following data repository. Repository name: Mendeley Data Data identification number: 10.17632/pfxd9j49xg.4 Direct URL to data: https://data.mendeley.com/datasets/pfxd9j49xg/4
Related research article	Authors' names: Arindam RoyChoudhury PhD, Thuy-Tien L. Dam MD, Chenghuiyun Xu MS, Jonathan H Diah MPH, Deepa Chaganty BA, Jonathan Solares BS, Linda P. Fried MD, MPH Title: Feed-forward Loop between Body Composition, Strength and Performance in Older Adults [3] Journal: Mechanism of Aging and Development DOI: 10.1016/j.mad.2019.111130

**Table:** 

**Value of the Data** • This dataset consists of measurements of body composition, strength and performance over a period of 12 years for 1466 subjects. Analysing these data may offer valuable insight on the process of aging as reflected in body composition, strength and performance. • Medical researchers and biostatisticians who are studying the association between body composition, strength, physical performance, and their effects on aging may benefit from the data. • New statistical models can be developed to reveal new relationships between the raw and constructed measures in the data, as well as how these relationships influence aging process and frailty. These models could be the first steps towards creating a system map of the development of frailty, leading to new interventions for treatment or reversal of frailty.

## 1 Data

The dataset consists of measurements from 1466 subjects. The first column lists non-identifiable Subject ID; Columns 2–16 list demographic variables, physical characteristics and medical history of the subjects (sex, diabetes status, hypertension, history of cancer, history of bypass surgery, weekly alcohol consumption, weight, height, history of stroke, smoking status, age, BMI, handedness, history of pain, history of surgery); Columns 17–33 list measures of physical performance and strength (ability to perform single-chair stand, limping, ability to perform tandem-walking, timed-up-and-go time, grip strength); Columns 34–63 lists measures of body composition (subtotal lean mass, total fat mass; trunk fat mass, subtotal fat mass, appendicular lean mass, appendicular fat mass, appendicular lean/fat ratio; log_2_-scaled subtotal lean/fat ratio).

Table 1, Table 2, Table 3, Table 4 present the result of feed-forward loop analysis on appendicular lean mass (ALM), grip strength (GS), subtotal lean mass/fat ratio (LFR), and timed-up-and-go time (TUG). Table 1 presents the parameter estimates and p-values for a feed-forward loop analysis between ALM and GS. Table 2 presents the parameter estimates and p-values for a feed-forward loop analysis between LFR and GS. Table 3 presents the parameter estimates and p-values for a feed-forward loop analysis between ALM, TUG and GS. Table 4 presents the parameter estimates and p-values for a feed-forward loop analysis between LFR, TUG and GS.

**Table 1:** Appendicular lean mass → grip strength → appendicular lean mass.

appendicular lean mass (g) → grip strength (kg) → appendicular lean mass (g)					
appendicular lean mass (ALM) → grip strength (GS)			grip strength (GS) → appendicular lean mass (ALM)		
GS_t+1_ = β_A→G_ ALM_t_ + β_G→G_ GS_t_			ALM_t+1_ = β_A→A_ ALM_t_ + β_G→A_ GS_t_		
	Estimate ± SE	p-value		Estimate ± SE	p-value
β_A→G_	0.14 ± 0.01	<0.001	β_A→A_	0.79 ± 0.01	<0.001
β_G→G_	0.81 ± 0.01	<0.001	β_G→A_	0.13 ± 0.01	<0.001

**Table 2:** Lean mass/fat ratio → grip strength → lean mass/fat ratio.

lean mass/fat ratio (log_2_) → grip strength (kg) → lean mass/fat ratio (log_2_)					
lean mass/fat ratio (LFR) → grip strength (GS)			grip strength (GS) → lean mass/fat ratio (LFR)		
GS_t+1_ = β_L→G_ log_2_ (LFR_t_) + β_G→G_ GS_t_			log_2_ (LFR_t+1_) = β_L→L_ log_2_ (LFR_t_) + β_G→L_ GS_t_		
	Estimate ± SE	p-value		Estimate ± SE	p-value
β_L→G_	0.03 ± 0.01	<0.001	β_L→L_	0.92 ± 0.01	<0.001
β_G→G_	0.92 ± 0.01	<0.001	β_G→L_	0.03 ± 0.01	<0.001

**Table 3:** Appendicular lean mass → up & go time → grip strength → appendicular lean mass.

appendicular lean mass (g) → up & go time (sec) → grip strength (kg) → appendicular lean mass (g)								
appendicular lean mass (ALM) →timed up & go time (TUG)			timed up & go time (TUG) →grip strength (GS)			grip strength (GS) →appendicular lean mass (ALM)		
TUG_t+1_ = β_A→U_ ALM_t_ + β_U→U_ TUG_t_			GS_t+1_ = β_U→G_ TUG_t_ + β_G→G_ GS_t_			ALM_t+1_ = β_A→A_ ALM_t_ + β_G→A_ GS_t_		
	Estimate ± SE	p-value		Estimate ± SE	p-value		Estimate ± SE	p-value
β_A→U_	−0.14 ± 0.01	<0.001	β_U→G_	−0.04 ± 0.01	<0.001	β_A→A_	0.79 ± 0.01	<0.001
β_U→U_	0.58 ± 0.01	<0.001	β_G→G_	0.90 ± 0.01	<0.001	β_G→A_	0.13 ± 0.01	<0.001

**Table 4:** Lean mass/fat ratio → up & go time → grip strength → lean mass/fat ratio.

lean mass/fat ratio (log_2_) → up & go time (sec) → grip strength (kg) → lean mass/fat ratio (log_2_)								
lean mass/fat ratio (LFR) →timed up & go time (TUG)			timed up & go time (TUG) →grip strength (GS)			grip strength (GS) →lean mass/fat ratio (LFR)		
TUG_t+1_ = β_L→U_ log_2_ (LFR_t_) + β_U→U_ TUG_t_			GS_t+1_ = β_U→G_ TUG_t_ + β_G→G_ GS_t_			log_2_ (LFR_t+1_) = β_L→L_ log_2_ (LFR_t_) + β_G→L_ GS_t_		
	Estimate ± SE	p-value		Estimate ± SE	p-value		Estimate ± SE	p-value
β_L→U_	−0.08 ± 0.02	<0.001	β_U→G_	−0.04 ± 0.01	<0.001	β_L→L_	0.91 ± 0.01	<0.001
β_U→U_	0.47 ± 0.02	<0.001	β_G→G_	0.90 ± 0.01	<0.001	β_G→L_	0.03 ± 0.01	<0.001

## 2 Experimental design, materials, and methods

### 2.1 Experimental design

All community dwelling adults living in Rancho Bernardo, California during 1972–1974 were eligible to participate [1]. For each participants, demographic information, physical characteristics, personal and family medical and health history were collected in the first visit. Some of the participants returned for subsequent visits approximately every four years [2]. This dataset consists of some measures collected in Visits 7–10.

### 2.2 Materials and methods

There were 1466 subject with at least one measure taken during visits 7–10. The current dataset presents raw and processed measures of body composition, grip strength and physical performance for these subjects.

Body composition was measured by fan-beamed dual energy x-ray absorptiometry (DXA) with the model Hologic 2000 (Hologic, Inc., Bedford, MA, USA).

Grip strength was measured by handheld dynamometer (Sammons Preston Rolyan, Bolingbrook, IL, USA). For each subject, grip strength was measured twice for each hand; the maximum value for each hand was recorded. The current dataset only contains the average of the maximum grip strength of the two hands.

For the timing in Time “Up and Go” Test (TUG), the subjects were asked to stand up from a chair, walk 3 m, and return back to the chair, to a sitting position. Each subject performed this test twice, and the times were recorded. The current dataset only contains the average of the two times.

### 2.3 Statistical modeling

We used two- and three-variable feed-forward loop models to analyze the feed-forward loop relationships between grip strength, appendicular lean mass, subtotal lean/fat ratio, and TUG. Here is a description of the models, which were fitted using the R package “sem”. Each variable was standardized separately for each visit, before the analyses were performed.

Following [4], the series of two variables {(X_t_, Y_t_)} is said to have a feed-forward loop relationship (with X- > Y- > X) if all three of the following conditions are satisfied.

X_t+1_ = β_11_ X_t_ + β_12_ Y_t_ + ε_1t_

Y_t+1_ = β_21_ X_t_ + β_22_ Y_t_ + ε_2t_

t = 1, … ,T − 1, where, β_kl_ (k = 1, 2, l = 1, 2) are parameters with β_12_, β_21_≠ 0, and ε_t_= (ε_1t_, ε_2t_) are independent and identically distributed (iid) bivariate random variables. No intercept term is used because that would introduce a trend among (X_t_, Y_t_). The outcome variables are represented by X_t_ and Y_t_, with (X_1_, Y_1_), (X_2_, Y_2_), … , (X_T_, Y_T_), representing measurement for T consecutive time points. Thus, each subject has a set of observations ((X_1_, Y_1_), (X_2_, Y_2_), … , (X_T_, Y_T_)). The data from n of such subjects may be represented as ((X_i1_, Y_i1_), (X_i2_, Y_i2_), … , (X_iT_, Y_iT_), i = 1,2, … n). If both of β_12_ and β_21_ are significant, then the existence of a feed-forward loop will be concluded.

We generalize this concept to three-variable loop model. The series of three variables {(X_t_, Y_t_, Z_t_)} is said to have a feed-forward loop relationship (with X- > Y- > Z- > X) if all three of the following conditions are satisfied.

X_t+1_ = β_11_ X_t_ + β_13_ Z_t_ + ε_1t_

Y_t+1_ = β_21_ X_t_ + β_22_ Y_t_ + ε_2t_

Z_t+1_ = β_32_ Y_t_ + β_33_ Z_t_ + ε_3t_

t = 1, … ,T − 1, where, β_kl_ (k = 1, 2,3, l = 1, 2,3) are parameters with β_13_, β_21_, β_32_ ≠ 0, and ε_t_= (ε_1t_, ε_2t_, ε_3t_) are independent and identically distributed (iid) trivariate random variables. No intercept term is used because that would introduce a trend among (X_t_, Y_t_, Z_t_). The data from n subjects may be represented as ((X_i1_, Y_i1_, Z_i1_), (X_i2_, Y_i2_, Z_i2_), … , (X_iT_, Y_iT_, Z_iT_)), i = 1,2, … n. If all three of β_13_, β_21_ and β_32_ are significant, then the existence of a feed-forward loop will be concluded.
